# Intelligent Sensors Security

**DOI:** 10.3390/s100100822

**Published:** 2010-01-22

**Authors:** Andrzej Bialas

**Affiliations:** Institute of Innovative Technologies EMAG, 40-189 Katowice, ul. Leopolda 31, Poland; E-Mail: a.bialas@emag.pl; Tel.: +48-32-2007-700; Fax: +48-32-2007-701

**Keywords:** Common Criteria, IT security development, intelligent sensor, design pattern

## Abstract

The paper is focused on the security issues of sensors provided with processors and software and used for high-risk applications. Common IT related threats may cause serious consequences for sensor system users. To improve their robustness, sensor systems should be developed in a restricted way that would provide them with assurance. One assurance creation methodology is Common Criteria (ISO/IEC 15408) used for IT products and systems. The paper begins with a primer on the Common Criteria, and then a general security model of the intelligent sensor as an IT product is discussed. The paper presents how the security problem of the intelligent sensor is defined and solved. The contribution of the paper is to provide Common Criteria (CC) related security design patterns and to improve the effectiveness of the sensor development process.

## 1. Introduction

The paper discusses the application of the ISO/IEC 15408 Common Criteria (CC) assurance methodology [1] to the development of intelligent sensors. Assurance is understood as a situation when an entity, e.g., designed IT product or system, meets its security objectives, in other words, the implemented measures will be able to counter threats when they occur. The paper presents a specific security view of a group of numerous and diversified technical devices called intelligent sensors, which contains sensor-, processing- and communicating facilities. Generally, sensors are devices that measure some physical quantity and convert the result into a signal which can be read by an observer or instrument, but intelligent sensors are also able to process measured values. A number of such devices contain actuators as well.

Intelligent sensors are used in many application domains, and the number of these domains is growing, though the number of finalized Common Criteria certification processes of these IT products is relatively low. The motivation of this paper is to provide developers with knowledge and specific design patterns related to the application of the Common Criteria methodology to the development of intelligent sensors. This can help them in a broader use of this methodology in dependable sensor solutions.

Generally, security design patterns are reusable proven solutions to security problems with respect to the specific context, and they are usually related to the software development, e.g., as discussed in the book [2]. These solutions may concern different issues: how to design identification and authentication, access control based e.g., on passwords, how to implement an encrypted communication channel, communication path, packet filtering for a firewall, accountability subsystem, *etc*. With respect to the Common Criteria methodology these security design patterns are related to the security functions implemented within the IT product or system according to the security functional requirements. Another kind of security design patterns is discussed in this paper—patterns closely related to the Common Criteria methodology, expressing: assets, legal subjects, attackers, threats, security policies, assumptions and security objectives, used to elaborate the security requirements specification for the IT product or system, and finally, the security functions implemented in the IT product or system. Here discussed “Common Criteria (CC) related security design patterns”, mentioned briefly in the paper as “patterns” or “design patterns”, are focused on the risk management issues, not on the IT security solutions. The CC-related patterns concern the design process (called here IT security development), can be used in many projects (*i.e.*, they are reusable), but in a certain context (*i.e.*, in the CC-methodology context). Such patterns can be applied for different kinds of IT products or systems but in the paper only the patterns subset related to sensors and sensor networks are discussed.

The paper focuses on the identification of intelligent sensors common features, allowing us to define a generalized model of such devices and, on this basis, to elaborate CC-related design patterns which can be used to specify security models of intelligent sensors. The discussed patterns concern only two stages of the Common Criteria compliant IT security development but these stages are of key importance: the security problem definition and its solution.

The paper includes the following sections. Section 2 contains a review of intelligent sensor security issues and sensor applications. Section 3 contains the CC methodology primer for sensor systems developers not familiar with this methodology. A generalized model of the intelligent sensor allowing to create the CC security model for itself is discussed in Section 4. Section 5 presents selected issues of the Common Criteria compliant development process applied to intelligent sensors, focusing on two important issues: specification of the security problem definition and solution. The proposed specification means were elaborated on the basis of a review of the literature included in Section 2. Section 6 discusses model evaluation. The last section concludes the paper and specifies the planned works.

## 2. Intelligent Sensors and Their Basic Security Issues

The progress in low-power CMOS processing, communication circuits and transducer technology has enabled new possibilities and applications, including advanced processing within the nodes (“motes”) of wireless sensor networks (WSNs). Apart from a class of sensors working in WSNs, other groups exist in different application domains. Intelligent sensors can work autonomously or co-operate with control systems supervised by SCADA or different monitoring units. Some of them are designed for special applications, e.g., motion sensors for digital tachographs.

The security and safety issues are important for intelligent sensors because very often they are used for responsible technical applications in varied, high-risk environments. These issues can be considered in the context of connectivity aspects due to the fact that intelligent sensors are elements of wireless sensor networks or can communicate indirectly with other systems in a different way. Basic research fields concerning sensor systems and their applications deal with energy efficiency, network protocols, distributed databases, as well as discussed here security issues.

Sensor systems designers focus on solving separate technical issues which are specific due to the resource limitation of the sensor nodes (limitations concern power sources, processing and communication capabilities).

The security analysis of wireless sensor networks [3] points at the possibility of individual node interception. This problem can be solved by using advanced cryptographic and authentication algorithms which require increased processing resources. The data transmitted between nodes and data stored within nodes should be secured. An important issue is to protect a cryptographic key used for securing data stored within nodes.

The denial-of-service attacks vulnerability of large scale sensor networks is discussed in the dissertation [4]. These attacks, performed from the outside or from an intercepted node, have different forms, such as: false data reports causing false alarms, excessive power consumption, and congestion of wireless channels. Due to the node computational limitation, some filtering algorithms, such as Bloom filters, are considered to solve this problem (messages can be broadcast to the selected nodes only, determined by a filter).

The paper [5] discusses basic security issues of wireless sensor networks, including the routing behaviour, node tamper resistance, and implementation of commonly known security mechanisms (encryption, key management, authentication, authorization). The use of these mechanisms is very difficult due to the node resource limitations. Specific solutions for wireless sensor networks have been developed based on the set of nodes provided with sensors and with actuators. The paper [5] presents an ambient intelligence application to control the environment. Environmental data are sampled by sensors, then aggregated and processed by external servers to extract knowledge about the environment. On the basis of this knowledge, control data are elaborated and passed to actuators. The data influence environmental parameters. The problem of data reliability has been solved on the application level with the use of a trust-based decision framework. The paper discusses most of commonly known attacks on the nodes, networks and applications, which will be considered later, once the intelligent sensor security model has been worked out.

A general review of security problems related to wireless sensor networks is provided in [6] with respect to both variants of networks: the hierarchical WSN (nodes of different capabilities, the network topology is known) and the distributed WSN (no fixed infrastructure, huge number of nodes, usually scattered in a hostile uncontrolled environment, the network topology is unknown). The paper studies well known WSN dedicated security protocols and solutions (SNEP—Secure Network Encryption Protocol, μTESLA—Micro Timed Efficient Stream Loss-tolerant Authentication, TinySec—the link layer security architecture for WSN, TinyPK—public key technology for WSN) which can be helpful in the implementation of the further discussed security objectives (Section 5).

The specific key management issues in a decentralized wireless network environment are discussed in the paper [7]. Different key management schemes with respect to network architecture and protocols properties have been developed, including NRFP (Novel Re-keying Function Protocol) presented in the paper. The network base station provided with long-lasting power is distinguished. It works as a controller (or a key server) for other nodes. The protocol is based on the symmetric key cryptography, hash functions and the used two modes of re-keying operations.

The paper [8] discusses the security problems specific to the Vehicular *Ad Hoc* Networks (VANETs) used to build applications improving road safety and providing information services for drivers. VANETs are a kind of MANETs, *i.e.*, Mobile *Ad Hoc* Network, which are self configuring networks of mobile devices connected by wireless links. In VANETs, road vehicles are equipped with network devices playing the role of routers. The communication is assured among nearby vehicles and between vehicles and nearby fixed equipment (roadside equipment). When a vehicle moves with high velocity, the topology changes rapidly. Due to this fact the following attacks can be performed more easily: jamming, data forgery, impersonation, and privacy violation. This may cause more complicated security problems. For example, issuing false car position information, or claiming multiple falsified identities (a Sybil attack) causing the illusion of traffic congestion, may cause serious traffic control system disturbance. To solve these problems, specific measures are used. VANETs security needs further researches and can be another field of applications of the presented method.

The paper [9] reviews the fourth generation of cellular communication systems (4G), the MANET networks and their common use in battlefield applications. The problem is to provide energy to such mobile devices. The features of both rechargeable and non-rechargeable batteries are discussed with respect to the battlefield requirements. The batteries have limited life, and their considerable weight (with reserve ones) should not reduce the soldiers’ mobility. Geographical factors (shape of the land) and environmental factors (natural mountains differ from urban areas with metal-concrete and electric barriers) may influence the communication ability of the military 4G MANETs. Additionally, specific risks related to the enemy’s actions disturbing communication between army forces should be considered during the development of these military wireless networks.

The specific issues of a mote-based medical sensor network are discussed in [10]. Long-term monitoring of vital signs of chronic-diseases patients requires dependable operations, data privacy and authenticity protection. The proposed security mechanism consists of a scheme for verifying the authenticity of the patient’s data, key agreement protocol to provide shared keys for sensor nodes and base stations, and the implementation of a symmetric key encryption/decryption system for protecting data confidentiality and integrity. Two hardware solutions for motes implementation were considered. One of them will be discussed later, during the identification of the common features of intelligent sensors and working out the security model for them.

The paper [11] summarizes the comparative analysis of common threats, vulnerabilities, attacks and applied measures for two areas: smart card/RFID technology and wireless sensor network node technology. The assumed TVAC security model (Threat-Vulnerability-Attacker-Countermeasure) can be useful to define properly threat items in the methodology presented below. While comparing these two technologies, it was pointed out that wireless sensor networks are poorly standardized. Besides, only few CC certificates concern the WSN technology. Wireless sensors developers can benefit from numerous experiences (hundreds of finalized CC certification processes) in smart card and RFID systems development because attacks on smart cards apply to WSN nodes and some node RF/Communications attacks may apply to contactless smart cards and RFIDs. Microcontroller architectures, tamper resistance, resource limitation, and communication issues are very similar in both technologies.

Intelligent sensors are able to work autonomously as well. The advanced Dräger Polytron 7000 Fixed gas detector [12] is an example of an intelligent sensor designed for responsible applications. It is able to detect over 100 different gases and has a modular structure. It is provided with communication facilities.

The implementation of the above mentioned mechanisms, like: cryptographic or filtering algorithms, advanced specialized key management or routing protocols in sensor networks, requires right assurance because they are extremely important to provide reliable and secure operation of the entire network and network-based applications. The above mentioned works focus on specific and advanced security mechanisms, however, they do not provide a methodology how to implement these mechanisms with proper assurance, for example with the use of well known assurance methodologies, like the ISO/IEC Common Criteria methodology. Considering the intelligent sensor as a typical IT product and the networked sensor system as an IT system, the Common Criteria methodology can be applied to them as well.

The works dealing with the Common Criteria application in sensors and sensor systems development are scarce, though they are becoming a new, promising Common Criteria standard application domain. The most relevant concern:

- airplane health monitoring systems,
- safety-critical assets distribution systems,
- motion sensors of digital tachographs,
- SCADA-related products,
- specialized firewalls used in control and automation systems, co-operating with sensor networks.

The paper [13] presents a sensor system, based on a wireless network, supporting the transfer of sensor data and information onboard commercial airplanes (eEnabled airplanes) as well as between airplanes and their supporting ground systems. Such systems, called AHMMS (Airplane Health Monitoring and Management Systems), are applied in the latest generation of aircrafts to improve the safety and efficiency of air travel. AHMMS monitor permanently the health of airplane structures and board systems using embedded sensors and give timely feedback to the flight control computer working on the board and to the airline ground server for health assessment. To secure the electronic distribution of software, cryptographic key and data between airplane and ground systems the Common Criteria methodology is used. The new application of this methodology encompasses the identification of specific security threats, requirements, and mitigation mechanisms based on the Public Key Infrastructure (PKI) services and digital signatures. Securing the communication channel between the aircraft and ground system is a very important issue, though it does not concern intelligent sensors directly, but communication with the sensor-based system.

A similar issue concerns asset distribution systems which should securely load the authorized software (e.g., safety standards compliant software, firmware for intelligent sensors) or contents (data, security related data, unique identifiers, cryptographic keys, *etc*.) to physical objects, like airplanes [14], vehicles or other specialized equipment. The integrity and authenticity of the distributed assets is significant for the safety and security of these objects. The presented research is focused on IT infrastructure for distribution of safety-critical and business-critical airplane software (EDS—Electronic Distribution of Loadable Software) and data [15].

In the automotive industry the assets can be loaded to different embedded electronic control units in vehicles [16], tachographs, their motion sensors [17], *etc*. While continuing research on cyber-physical systems and eEnabled applications, some challenges were identified [16], like: building of specialized high-assurance PKI, use of formal methods for an end-to-end analysis of assets distribution systems, removing vulnerabilities and analyzing the impact of security on safety. The Common Criteria methodology can effectively support these efforts.

The digital tachograph system [17–19] consists of a vehicle unit, the considered motion sensor and a smart card used to log in to this unit. The uniquely identified motion sensor includes a dedicated microcontroller placed, together with other circuits, on a relatively small sized printed circuit board—located in a sealed housing, which is screwed into the gear box. The movement detector (a hallotron) transforms the rotations to electric pulses which are sent to the microcontroller of the vehicle unit as a rough “Speed signal” and to the motion sensor microcontroller. The data are processed concurrently in both locations and results are compared. The microcontroller exchanges information (distance travelled, speed, status, commands) with the vehicle unit through a cryptographically secured channel (“Data signal”). The motion sensor performs advanced data processing in a heavy duty environment. The Common Criteria standard is used to evaluate the security of tachograph systems [20–22]. For the motion sensors, a full life-cycle model was applied, considering not only the operational environment, but also development-, manufacturing- and maintenance environments. This means, for example, that the threats existing in the development environment, e.g., “breaching design data”, “improper testing” are considered because they may influence the motion sensor misbehaviour in its operational environment.

Intelligent sensors can be used in control systems (field controllers) cooperating with SCADA as well. Security problems of these systems are growing because they are often parts of critical information infrastructures. The paper [23] discusses a new generation of SCADA systems used to control virtual utilities, aggregating distributed resources, like: microgrids, wind farms, fuel cells, *etc*. into single, centralized energy systems. Analyzing the security of these systems, the author of [23] concludes that SCADA products should be evaluated with the use of the Common Criteria standard to avoid compromising the security or safety of the critical infrastructure by these products.

Moreover, the Common Criteria standard was applied in the development of the equipment used to secure wireless networks, like firewalls, routers, intrusion detection systems, and the microcontrollers to be used for intelligent sensors implementation, including control automation.

Analyzing these works it is possible to create a common picture related to the intelligent sensors security features, applications and sensors co-operation within other systems. It should be also stressed that there is lack of knowledge and CC-related design patterns possible to use in the IT security development. The Common Criteria methodology requires a specific, methodical approach to the identification and description of security items, like threats, security policies, countermeasures, *etc*.

The works discussing the character of known attacks [3–9] do not present them with the use of the CC methodology. Still, these works can be helpful to define these attacks according to the CC specification convention (*i.e.*, attacker-asset-attack method/ used vulnerability) to define measures countering these threats and to provide the required rationale for the applied measures.

The paper [11] points at similarities between the smart card/RFID technology and wireless sensor network node technology with respect to threats, vulnerabilities, attacks and applied measures. Because the smart card/RFID solutions group is one of the most numerous groups of certified IT products or systems [24], the published security specifications for these solutions can be helpful for wireless sensor developers as well as with respect to the above mentioned risk management issues, *i.e.*, threats, vulnerabilities, *etc*.

The complete set of specification items for the motion sensor of digital tachographs is published in [17] but it is specified by an old EU standard (ITSEC – Information Technology Security Evaluation Criteria, 1991). These items, called also “generics”, can be used indirectly for motion sensors evaluated according to Common Criteria, like the sensor described in [20]. Some other works [13–16, 23] have focused on securing systems co-operating with sensors systems are emerging and do not concern intelligent sensors directly, but rather their environment.

To sum up, there is little research focused directly on the intelligent sensors development according to Common Criteria. Some works deal with platforms for experimentations built to enable sensors applications in high-risk environments. In this case the security issues do not go beyond the operational environment and full life-cycle model, encompassing also the development, manufacturing and maintenance environments. The intelligent sensor development can be discussed as one of the emerging application domains of the CC standard. Similarly to other new domains, it requires support for IT security developers in order to facilitate their work. This support may concern Common Criteria related security design patterns, knowledge on how to use these patterns and specialized tools.

## 3. Common Criteria Methodology—Primer

The Common Criteria standard (ISO/IEC 15408) is the leading assurance methodology which provides dependable IT solutions for applications when the assessed risk is high or the appreciated asset value is significant. As it was mentioned above, assurance is the confidence that an IT product or system, called TOE (target of evaluation), meets the security objectives specified for it. This means that the built-in security functions related to these objectives and representing measures will work effectively whenever a threat occurs. Assurance is measured by evaluation assurance levels (in the range EAL1 to EAL7) and depends on the rigour applied to the security development.

The first part of the standard [1] presents a general model and structures of basic security requirements specifications, called security target (ST) and protection profile (PP). The second part includes functional components used to express security functional requirements (SFRs) for TOE security functions representing TOE built-in countermeasures. The third part contains assurance components used to express security assurance requirements (SARs) for these security functions. Both sets of components constitute a semiformal “language” to uniformly express security requirements.

The EALs are well balanced packages (sets of components) of SARs. Both kinds of CC components are grouped by families which, in turn, are grouped by classes. They are used as specification means of security requirements.

The assurance level is claimed by developers who provide evidences that the given EAL is met. An IT product or system (TOE), its security target (ST) and the related evidences are evaluated to get the certificate [24]. The ST is considered as a complete, implementation-dependent security specification of the TOE. The main parts of the ST are: security problem definition, security objectives, security requirements and security functions. The second kind of the security specification is the mplementation-independent protection profile (PP) which does not contain these functions. Security targets can be elaborated directly on the basis of the users’ requirements or with the use of previously evaluated protection profiles.

The CC methodology encompasses the following processes:

- IT security development process, identified with the security target (ST) work-out, specifying the TOE security functions;
- TOE development process with the use of the assumed technology (elaboration of the TOE evaluation evidences, expressing the implementation of these functions on the EAL declared for the TOE);
- IT security evaluation and certification process (not discussed here), providing independent analyses, verifications if the claimed EAL is met.

The IT security development process [1]/Part 1 has the following stages:

1. Work-out of the “ST introduction”, containing different identifiers, informal TOE overview and TOE description;
2. Conformance claims with the Common Criteria version, protection profiles and packages, including EAL;
3. Security problem definition (SPD); SPD specified threats, OSPs (organizational security policies) and assumptions for the TOE and its environment;
4. Solution of this problem by specifying security objectives (SO)—for the TOE and its environment;
5. Extended components definition, when needed (*i.e.*, when the developer should define his/her own component due to its lack in the standard);
6. Work-out of the security functional requirements (SFRs) specification on the security objectives basis, and the set of security assurance requirements (SARs) which are derived mainly from the declared EAL;
7. Creating the TOE summary specification, including the TOE security functions derived from the SFRs; they are implemented in the TOE on the claimed EAL during the TOE development process.

The TOE development is the second CC key process. Apart from the security target itself, the mentioned evaluation evidences encompass documentation and records concerning ([1]/Part3):

- TOE architecture, its functional specification, design, security policy, implementation—expressed by the ADV (Development) assurance class components (please note that according to [1] each assurance class name begins with the letter “A”, two other letters concern the issues described by the class),
- configuration management, product delivery, development process security, used tools—represented by the ALC (Life-cycle support) class components,
- tests specification, test depth and coverage – influenced by the ATE (Tests) class components,
- product manuals and procedures, worked out according to the AGD (Guidance documents) class components,
- vulnerability assessment according to the AVA (Vulnerability assessment) assurance class.

When IT products or systems are properly designed, managed in a life cycle, fully and deeply tested, carefully documented, with identified and assessed vulnerabilities, and, moreover, thoroughly evaluated by an independent body, they can be applied in high-risk environments.

## 4. Intelligent Sensors Model for IT Security Development Process

The paper is focused on intelligent sensors (sensor nodes, motes), not on sensor networks or control- and SCADA systems, although connectivity aspects will be considered too. To discuss the security features of the intelligent sensor, a common, generalized sensor model should be elaborated. It will be used for security consideration according to the Common Criteria methodology. To develop the generalized intelligent sensor model, another group of publications, presenting different sensors architectures, ought to be reviewed.

The paper [25] discusses the architecture and features of wireless intelligent sensors with complete systems on a chip, integrated low-power communication facility, and integrated low-power transducers. The presented example of an intelligent sensor, used to measure temperature and light, consists of a microcontroller (MCU) with internal memories: flash program memory, data SRAM and data EEPROM. It is connected to a set of sensors and actuators. The intelligent sensor also includes LEDs (connected to I/O ports displaying digital values or status), a low-power radio transceiver (for external communication), analogue photo-sensor, digital temperature sensor (A/D converter connected by means of I^2^C, *i.e.*, Inter-Integrated Circuit interface), serial port (UART), and a small coprocessor unit. This unit is connected by SPI, *i.e.*, Serial Peripheral Interface, to allow sensor reprogramming by transferring data from the network into the coprocessor EEPROM. As an operating system, TinyOS is applied allowing concurrency handling in a very limited memory space. Because the presented intelligent sensor is a prototype for evaluation and further development, physical security (a case, seal, *etc*.) was not considered for it. The device is simply implemented on a small printed circuit board, convenient for experimentation.

Hardware solutions for sensor nodes (called motes) used for permanent monitoring of long-term care patients are presented in [10]. One of the used hardware platforms was Crossbow MICA [26]. MICA (as well as IRIS), operating on TinyOS, consists of: Atmel© ATMega128 MCU (8-bit microcontroller with 128 K Bytes In-System Programmable Flash, digital and analog I/O) [27], RF transceiver, flash data logger memory (storing data and measurements, over-the-air reprogramming under TinyOS), power source (batteries), and sensor board interface (to connect peripheral sensors and data acquisition boards or base station, including connections for: A/D converter, UARTs, I^2^C, digital I/Os). The latest IRIS is equipped with the ZigBee standard [28], ready radio frequency transceiver (of better range), and sensor ID facilities. Many advanced solutions were developed according to this proprietary standard, including network processors [29] with implemented protocols.

The paper [30] describes another new general-purpose wireless sensor node architecture designed for experimentation, educational projects and preliminary deployment in industrial environments. It is based on the OKI ARM ML67Q500x microcontroller and Chipcon CC2420 ZigBee-compliant communication subsystem. Four-layer software architecture was applied, encompassing: sensor drivers, preprocessing and measurement storage, localization algorithms, and sensor network applications.

Echelon FT 5000 Smart Transceivers/ Neuron®5000 [31] is another representative example of a platform for intelligent sensors. The Neuron chip series, provided with multiple processors, ROM, RAM, communication and I/O subsystems, is able to perform key functions necessary to process inputs from sensors and control devices, and to propagate control information across different kinds of network media. Neuron contains three processors to manage the operations of the chip, the network, and the user application. At higher clock rates, it is additionally equipped with a separate processor to handle interrupts. Peripheral transducers (sensors, control devices) can be connected through I/O subsystems. The SPI and I^2^C interfaces can be used for external coprocessor or memory (EPROM, FLASH) connection. The above mentioned FT 5000 transceiver, connected to the Neuron communication subsystem, is designed for the polarity-insensitive, free-topology, twisted-pair LonWorks networks. Each chip has a unique 48-bit identification number, called Neuron ID. Related Neuron firmware provides implementation of the LonTalk® protocol for LonWorks networks, developed by Echelon for years.

**Figure 1.**
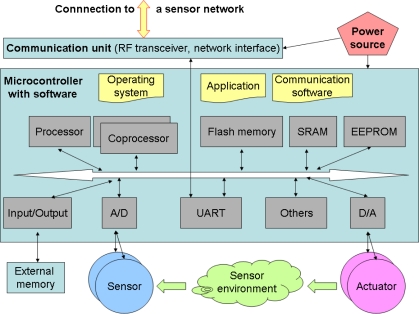
Generalized model of the intelligent sensor used for security analyses during the IT security development process.

The review shows that intelligent sensors have similar architecture. Analyzing these selected solutions, a general model has been elaborated. The model serves as a reference model for the devices discussed here. Sometimes refined, it can be used to prepare the “TOE description” being a part of the security target specification, to define the security problem and to propose its solution. The level of details should be adequate to perform these tasks – a block scheme level is usually enough. A simplified, generalized sensor model, co-operating with the sensor network and elaborated on the basis of the above examples, consists of four-key elements (Figure 1):

- microcontroller, equipped with a specialized operating system, communication and application software, different devices, such as memories, including non-volatile memories, interfaces, timers, counters, sometimes other coprocessors (e.g., crypto-processors, network processors and other specialized processors), interfaces to external memories, and other devices (I^2^C, SPI),
- low-power transducers (varied sensing/controlling devices),
- communication facilities, including low-power wireless facilities or traditional/industrial network interfaces (wired, optical facilities),
- power source (battery, solar, external sources).

The intelligent sensor is able to perform some processing tasks to sample information and to communicate with other connected nodes in the network. Restricted sensor resources (related to processing, transmission, energy) limit its capability.

The general model should be able to express the TOE user functionality and the essential parts of the TOE with respect to security issues, such as: what is a part of the TOE and what is a part of the TOE environment, connections with co-operating entities, logical and physical TOE boundaries, *etc*. These issues are important when the above mentioned TOE overview and TOE description sections of the security target are developed. Moreover, the general model should contain details sufficient to identify protected assets (where they are, who their owners are), affecting intruders, entities co-operating with the TOE, threats, and certain assumptions (e.g., dealing with the connectivity aspects). These issues are considered during further discussed security problem definition and solution.

Depending on the project character, the TOE may encompass almost all elements from Figure 1, or only its selected parts, e.g., the TOE may be a “microcontroller without coprocessor”, “(cryptographic) coprocessor of the microcontroller”, “communication software” or “application which processes, in the microcontroller, the data sampled by the sensor”, *etc*. Different solutions for different applications are possible. Analyzing the security of a given sensor node as the TOE, some details can be added, e.g., those from the above reviewed solutions.

## 5. Common Criteria IT Security Development Process—Intelligent Sensor Specific Issues

The IT security- and TOE development processes include different security analyses of interrelated issues, rationales, decisions, and work-out of extensive and precise documents. The mentioned processes are time-consuming and costly, require specialized knowledge and, basically, are difficult for IT developers, including electronic engineers. These problems disable a broader use of dependable IT solutions. The paper is related to the author’s more extensive works aiming at the improvement of the CC methodology by introducing a semiformal description and by using the knowledge engineering methodology.

The Common Criteria compliant, UML/OCL-based IT security development framework (ITSDF) [32–34] was elaborated. The framework embraces:

- models of data structures and processes of IT security development stages, including: security problem definition, security objectives elaboration, security requirements, and security functions workout;
- models of the specification means used for these IT security development stages, including CC components and the introduced semiformal enhanced generics.

The introduced enhanced generics, derived from “generics” commonly used by developers, are defined as mnemonic names expressing common features, behaviours or actions related to IT security issues, like: subjects, objects, threats, assumptions, security policies, security objectives, and functions. They are “enhanced” since they are semiformal and have features comparable to CC components, allowing such operations as: parameterization, derivation, iteration, and refinement. The semiformal ITSDF framework was implemented as a software tool to support IT security developers.

In the next step, the knowledge engineering methodology [35] was applied to this framework [36,37]. Using the Protégé tool from Stanford University [38], the domain ontology and related knowledge base were elaborated. Generally, ontology represents explicit formal specifications of the terms in the domain and relations between these terms. The considered domain is the discussed Common Criteria development methodology.

The elaborated ITSDO (IT Security Development Ontology) represents the security requirements structures (ST, PP), specification means to fill in these structures with contents for different TOEs (author’s defined enhanced generics, CC-defined functional and assurance components) as well as patterns for evidences. About 350 enhanced generics were predefined as elementary items designed to specify general security features of commonly used IT product or systems. The intelligent sensors discussed here are very specific, so their specification items have not been elaborated yet. This paper presents the first step of the work aiming at the extension of the elaborated ontology and knowledge base to the new field of applications, *i.e.*, intelligent sensors development. These devices can be considered as a certain knowledge subdomain extending the existing knowledge domain.

Due to the extended range of this work, it was divided into two tasks. The paper encompasses the first task – the identification of terms and relationships constituting the considered ontology subdomain. The identified below enhanced generics can be used as Common Criteria related security design patterns and to express security issues of any intelligent sensor in a traditional way (*i.e.*, without knowledge base support). Besides, defining these generics enables the knowledge base extension to intelligent sensors.

In the next task, planned in the near future, the identified terms and relations will get ontological representation providing new possibilities offered by the knowledge engineering approach, like [35]:

- sharing common understanding of the structure of information among people or software, *i.e.*, mainly the structure of the components, generics and evidences;
- reusing the domain knowledge, *i.e.*, using the same specification means in different projects and deriving their new variants from the previously defined ones;
- making explicit assumptions for a domain; it concerns predefined parameters and predefined mapping relations between specification items;
- separating the domain knowledge, expressed by the specification means as a whole, from the operational knowledge allowing to use these means to compose the ST of the given IT products or systems;
- providing the domain knowledge analyses concerning: variants, semantics, risk, relationships of the developed specification means, *etc*.

### 5.1. Defining Common Criteria related security design patterns—general rules

Enhanced generics can be used as predefined Common Criteria related security design patterns which allow one to compose IT security specifications for varied intelligent sensors. This subsection gives a short introduction to the assumed enhanced generics notation, which is similar to the notation commonly used by IT security developers (dot separated fields) but more precise. In the author’s previously mentioned works the enhanced generics were defined on a different level of abstraction, like formal grammar, UML objects, ontology classes individuals, sets of literals, *etc*. For this work it is enough to assume that, similarly to the SFR/SAR components, an enhanced generic consists of textual fields separated by dots:

Family.Mnemonic.Description.Refinement

The *Family* field groups similar items. Each security model issue, like: assets, subjects, threats, *etc.*, has its own possible values – mnemonic prefixes representing families.

According to the CC-defined functional paradigm [1]/Part 2, an asset represents a passive entity within the considered system. The *Family* field for assets (data, services, software, IT infrastructure, documents, *etc*.) begins with “D”, and the following values are allowed (the symbol “|” means “or”): *Family* ::= DTO|DIT|DAP, where:

“DTO” – “TOE related assets”,
“DIT” – “assets within the TOE IT environment”,
“DAP” – “assets within the TOE physical environment”.

Subjects, representing active entity, are preceded by prefixes: *Family* ::= SAU|SNA|SNH, where:

“SAU” – “authorized subject, e.g., user, administrator, process”,
“SNA” – “unauthorized entity, e.g., intruder”,
“SNH” – “non-human malicious entity, e.g., force majeure, failure”.

For threats, the following prefixes are defined: *Family* ::= TDA|TIT|TPH, where:

“TDA” – “direct attacks against the TOE”,
“TIT” – “attacks against the TOE IT environment”,
“TPH” – “attacks against the TOE physical environment”.

Assumptions, addressed to the TOE environment, have prefixes: *Family* ::= ACN|APR|APH, where:

“ACN” – “connectivity aspects”,
“APR” – “personnel/organizational aspects”,
“APH” – “physical aspects”.

Similar prefixes are defined for security objectives:

*Family* ::= OACC|OIDA|OADT|OINT|OAVB|OPRV|ODEX|OCON|OEIT|OEPH|OSMN and for organizational security policies (OSPs):

*Family* ::= PACC|PIDA|PADT|PINT|PAVB|PPRV|PDEX|PCON|PEIT|PEPH|PSMN, where:

“OACC/PACC” – “access control and information flow control”,
“OIDA/PIDA” – “identification and authentication”,
“OADT/PADT” – “accountability and security audit”,
“OINT/PINT” – “integrity”,
“OAVB/PAVB” – “availability”,
“OPRV/PPRV” – “privacy”,
“ODEX/PDEX” – “data exchange”,
“OCON/PCON” – “confidentiality”,
“OEIT/PEIT” – “TOE IT environment”,
“OEPH/PEPH” – “technical or infrastructure”,
“OSMN/PSMN” – “security maintenance”.

It should be mentioned that enhanced generics may express security functions as well (not discussed in the paper). Prefixes of the security functions begin with the letter “S”, and further include a CC functional class name which the given function deals with, e.g., for the “FAU” class (audit), the security functions have the “SFAU” prefix.

The second enhanced generics field, developers-defined *Mnemonic*, expresses as briefly as possible (a few letters) security features, behaviour or actions (see examples below).

The *Description* field presents concisely the generic meaning, using one or few sentences. This field may have parameters in square brackets representing any asset [*Dparam*] or any subject [*Sparam*]. Parameters may be left empty (meaning: “any possible”) or substituted (symbol: “<=”) by an appropriate asset or subject generic. Thanks to parameterization, the iteration of enhanced generics is possible. In this case the given generics can be placed many times into the specifications with different parameters substituted, and presenting different aspects of the same issue. For example, the threat item with intruders of different attack potential attacking the same asset, or the threat with one intruder attacking different assets in a different way. Particular instances of the iterated enhanced generic are numbered, placing their consecutive numbers in brackets. The content of the *Description* field is predefined for enhanced generics placed in a tool library or knowledge base. By using the given generics in the project, some extra information can be added on the project level by means of the last, optional field, called *Refinement*. Similarly to the refined CC components, this word is placed below the generic description as an underlined word *Refinement*, which will be shown below using examples.

### 5.2. Common Criteria related security design patterns for the security problem definition and solution by specifying the security objectives

This section presents examples of enhanced generics that can be used to compose the security problem definition (SPD) of the targets of evaluation which are intelligent sensors. Most of these generics can be applied for sensor networks as well. These generics are defined on the basis of the author’s earlier work [21] and the results of the analysis of varied specific attacks on wireless sensor networks (WSN) described in Section 2.

The depth of the analysis required to elaborate the security problem definition depends on the designers’ needs and design character. For most of the identified elementary security issues (threat, OSP, assumption), security objectives (SO) are proposed to solve these elementary problems. Some objectives can be specified for the TOE and some of its environment. The most representative specification items are shown below. They can be used by developers to elaborate the security problem definition and security objectives specification for the given TOE. The elaboration of these specifications constitutes two of key importance initial stages of the IT security development process. The proposed enhanced generics encompass a full life-cycle model, starting from the TOE development phase, through manufacturing-, maintenance-, operation phases until the end-of-life phase. This broader view of assets, actors, threats, policies, assumptions, *etc*. in the life cycle, is important especially for sites distributed and compliant with the recently developed site certification concept [39].

#### 5.2.1. Assets and other passive entities

Assets represent passive entities protected by the TOE, though some of them are placed in it and some are external. Enhanced generics are able to precisely express the identified assets. It should be known what is protected by the TOE. The value of an asset is important along with the risk (considered optionally) and they both influence the claimed EAL. Assets can be attacked by intruders, which is expressed by threats. Moreover, assets should be managed, which is expressed by the OSPs. For this reason the assets generics can be used as parameter values of other kinds of generics, especially threat and OSPs generics.

The basic asset for any intelligent sensor are data sampled for users and data related to the sensor operation (data processing) and communication. These data can be expressed using the following TOE-related enhanced generic:

DTO.SensorData. Data measured, stored, processed, or transmitted by the intelligent sensor and data related to the network services.

Usually the intelligent sensors have restricted resources. The availability of these resources allows right operation of the sensors. It is especially important for sensors which work within a wireless network. Sensor resources critical for the right operation can be expressed in the following way:

DTO.NodePowerRes. Energy supplying a sensor node.
DTO.NodeProcesRes. Node processing ability.
DTO.NodeTransmRes. Node data transmission ability.

Depending on the domain and character of application, sensors are provided with different security measures concerning: identification, authentication, protection of confidentiality or integrity, *etc*. Data related with these measures, called security-related data, are also assets requiring special protection, for example:

- DTO.SensorID. Unique identification number of an intelligent sensor.
- DTO.Password. Password for authentication.
- DTO.JoinKey. Keys allowing to connect to the network.
- DTO.CryptoKey. Symmetric cryptographic key.
- DTO.Credent. Credential or shared secret.
- DTO.RndNumber. Random number to derive a cryptographic key.
- DTO.PubKey. Public key.
- DTO.PrivKey. Private key.

To consider more complex relationships, like: mutual security dependencies, propagation/escalation effects, it is necessary to take into account other sensor nodes, computer systems and software applications existing in the network. These assets are placed within the TOE IT environment, which is denoted by the “DIT” Prefix. Moreover, the data sampled as a whole from all network sensors may be considered too.

DIT.BaseStation. A distinguished node of a wireless sensor network (WSN) used to control the network or as a gateway intermediating between WSN and other network.
DIT.CentralUnit. The entity supervising and/or monitoring sensors.
DIT.NeighbourSenNode. A neighbour node whose security mutually relies on the considered sensor node.
DIT.SampledDataBase. Data sampled by sensors and stored in a common data base on the distinguished server.

For certain kinds of sensors, when a detailed life-cycle model is applied, the assets related to the development-, manufacturing- and maintenance processes performed in the “site” [39] are considered. This is because breaching these assets influences indirectly the basic or security-related assets of sensors operating in the network. For example, an unauthorized modification of a firmware, configuration lists, tests, specialized equipment, options of compilers, *etc*. during the development/manufacturing process, may later cause serious problems in the operation environment. These assets exist far from the TOE operational environment—somewhere in the TOE physical environment, *i.e.*, in the “site”. All these assets can be expressed by one specification item of the “DAP” prefix, which can be also refined by adding some details (please note the refinement operation example).

DAP.DesignData. Sensitive project data and documentation.
Refinement: Logical, physical design data of the TOE hardware, software specifications, code and other related documentation, development aids, test data, user data related documentation, material for software development and manufacturing process.

#### 5.2.2. Subjects and other active entities

The next step is to identify active entities, including asset owners, legal users, intruders and other “actors” related to the TOE and its environment. The basic subject of the intelligent sensor system represents a “sensor user”, who/which can take different forms depending on the user’s nature. The personnel as the “sensor user” usually communicate with the sensor indirectly, through the base station and network where the sensor works. Sometimes the “sensor user” can be considered as a process communicating directly with the sensor to get some data or to perform some operations. In some conditions an authorized subject can play the role of an intruder as well. Moreover, the administrator of the network assets and applications is distinguished.

SAU.IntellSensorUser. Authorized entity (user, process) who/which directly or indirectly uses the intelligent sensor.
SAU.SensorNetAdmin. Authorized administrator of the sensor network and applications.

To reflect the personnel activities (legal, permitted and malicious) in development, manufacturing and maintenance processes, the following items representing key actors are predefined:

SAU.Developer. Personnel involved in the design phase (hardware/software designer, programmer, test engineer).
SAU.ManufPers. Personnel involved in the manufacturing processes (components manufacturing, assembly, security data insertion, storage, distribution, repair).
SAU.ServicePers. Personnel involved in sensor or sensors system maintenance (storage, installation, inspection, calibration, repair).

Intruders in the operational environment including initiators of harmful events are the following:

SNA.HighPotIntruder. Attacker having high level skills, enough resources and deep motivation to perform a deliberate attack.
SNH.ForceMajeure. Different kinds of unforeseen reasons of harmful events.
Refinement: Catastrophes, accidents, failures, overheating, fire, water, *etc*.

Intruders in the development-, manufacturing- and maintenance environment are specific:

SNA.IndustIntrud. Industry spy or intruder trying to get or counterfeit the design data.

#### 5.2.3. Threats related to the intelligent sensor with respect to the full life cycle

The predefined enhanced generics allow to specify varied threats. Most generics have parameters expressing active entities, e.g., legal subjects, intruders (Sparam), and passive entities, e.g., attacked assets (Dparam). Thanks to these parameterization- and refinement abilities, the iteration can be applied while the threats specification has been elaborated. As it was mentioned earlier, the iteration allows to place a given threat into the specification with different parameter values assigned, or with different refinements added. Different kinds of threats are distinguished. For any group of threats representing elementary security problems, two groups of related items are proposed:

- representative examples of passive and active entities that can be considered as threat parameter value,
- security objectives for the TOE and/or for its environment (lines indented) countering these threats and being the typical solutions of these elementary problems.

Sometimes one threat can be solved by one security objective, but usually some objectives are specified for the TOE and some, of supporting character, for the TOE environment. Below, one or more objectives are added for any group of threats (intended lines).

Basic threat items express security issues related with the operational environment, especially attacks focused on the data sampled, stored, processed and transmitted by the intelligent sensor. These items will be supplemented later by threats related to the development-, manufacturing- and maintenance-processes.

First, a threat example concerns a direct attack against data sampled (measured) by the sensor. This generic can be refined depending on the kind of sensor and measured data. The important issue is that input data cannot be disrupted or counterfeited by any reason. The proposed security objectives (countermeasures) concern tamper-resistance, reliable solutions and sensors inspections. For some operational environments, some of these measures can be impossible or hard to implement. Instead, the network redundancy mechanisms can be applied. For sensors provided with actuators, a very similar elementary security problem can be defined and solved with respect to the output data of the actuator.

TDA.DisruptSampling. Users or intruders [Sparam <= SAU.IntellSensorUser, SNA.HighPotIntrud] could try to manipulate the sensor input [Dparam <= DTO.SensorData] causing wrong input data.
  OINT.TamperResistance. The TOE guarantees its own physical/logical integrity. The means of detecting physical tampering must be provided (e.g., seals, tampering detection, special reinforced cases, intrinsically safe solutions).
  OAVB.Reliability. The sensor must provide reliable service. Applying fault tolerance methods and techniques.
  OSMN.RegularInpections. The sensor must be periodically inspected and calibrated (if necessary).
  OAVB.RedundNodes. Apply redundant sensor nodes, allowing to lose nodes without any impact on the network (or network application) behaviour as a whole.

The data properly sampled by the sensor are stored, processed and prepared for sending through the network to the central database or to any process control or monitoring unit. The second group of threats concerns direct attacks against the data stored or processed within the sensor node. The data can be eavesdropped, modified or even fabricated. It is possible due to simple or complex network attacks finalized by illegal data access. Depending on the node and threat character, different aspects should be emphasized in the threat item definition. The first example describes complex attack on the node from the network, three others—more specific attacks. To counter these threats, the access control, fault tolerance and authentication mechanisms are generally recommended. To protect confidentiality, cryptographic methods should be applied. All of them can be supported by audit facilities. The barrier of the node resources (discussed further) can be encountered by applying advanced cryptography.

TDA.NodeCompromise. Attacker [Sparam] can: eavesdrop the traffic, inject packets or replay older messages, because wireless communication generally is not secure. After the node compromising, all information it holds [Dparam] is known to the attacker and/or node operation or communication is broken [Dparam].
  OACC.Access. The sensor must control access of connected entities.
  OIDA.Authentication. The sensor must authenticate connected entities.
  OADT.Audit. The sensor must audit attempts to undermine its security and should trace them to the associated entities.
  OINT.Processing The sensor must ensure that the processing of input to derive output data [Dparam <= DTO.SensorData] is accurate.
  OAVB.DataFreshness. Ensure that the message received [Dparam] is the message sent by the authorized source [Sparam] but not a replayed message sent by the intruder [Sparam].
TDA.Access. Users or intruders [Sparam <= SAU.IntellSensorUser, SNA.HighPotIntrud] could try to access functions or data [Dparam] they are not allowed to.
  OACC.Access. The sensor must control access of connected entities.
TDA.SensorDataEavsdrop. Intruder [Sparam <= SNA.HighPotIntruder] eavesdrops sensor data [Dparam <= DTO.SensorData].
  OCON.DataEncrypt. Encrypt the data [Dparam <= DTO.SensorData].
TDA.SensDataMdfy. Intruder [Sparam <= SNA.HighPotIntruder] modifies sensor data (falsifies them) [Dparam <= DTO.SensorData].
  OIDA.Authentication. The sensor must authenticate connected entities.

The sampled and partially processed data are sent to other nodes, data base servers or to any control/monitoring equipment. The next group of threats represents attacks aimed at sensor networks and the sensor node transmission ability, constituting the TOE IT environment (TIT).

TIT.CommInterfer. Intruder [Sparam <= SNA.HighPotIntruder] interferes network communication by sending messages through different protocol layers (e.g., jamming, collisions, flooding), causing that the data [Dparam <= DTO.SensorData] are lost (usually inconspicuously for their owner or destination) or the node disappears (“has been stolen”).
  ODEX.CommQuality. Avoidance of interference, blocked communication spaces, using specialized measures against jamming, collision and flooding.
  OINT.DataVerification. Verify that the data [Dparam] are valid.
  OINT.MajorityVoting. Apply the majority voting scheme to determine the validity of an alarm raised by neighbouring nodes based on their own measurement.
TIT.RoutingMisuse. Intruder [Sparam <= SNA.HighPotIntruder] interferes routing by ignoring all or some messages or network communication.
  ODEX.MultipleCommPaths. Use redundant communication paths, specialized countermeasures (e.g., against blackholes, misdirection, wormholes), and controlling of the routing information.
TIT.Malware. Malicious software designed to infiltrate or damage a sensor node or sensor network.
TIT.BackdoorOpen. Using the wireless access point an intruder [Sparam <= SNA.HighPotIntruder] creates a backdoor for the network of an organization (corporation) from the sensor node side.
TIT.AttackPropagation. The possibility of attack propagation from a node to other nodes, gateways and external servers.
  OACC.Access. The sensor must control access of connected entities.
  OIDA.Authentication. The sensor must authenticate connected entities.
  OINT.AntiMalware. Specialized anti-malware software.
TIT.UncontrolledArea. Due to uncontrolled pervasiveness of a wireless sensors network, the node works in an uncontrolled network area accessible to potential intruders [Sparam <= SNA.HighPotIntruder].
  OEIT.SecPerimVsTrRangeCtrl. The physically controlled security perimeter, where nodes are placed, should be defined with respect to the range of wireless transmission.

For some safety-critical applications a special class of threats should be considered, which express scenarios how IT security problems may cause safety problems. This kind of threat has complex character just like the above mentioned TIT.CommInterfer and should be refined after a detailed analysis of the threat nature in the considered environment, e.g., in the aircraft environment. The selection of security objectives for it is not easy, because probably the multilayered protection should be applied, encompassing most of the above specified objectives.

TIT.SafetyProblem. The manipulation (corrupting, replaying, blocking) by [Sparam] of the data [Dparam] sampled by sensors with the intention of hiding or delaying detection of safety-critical faults in the safety critical equipment to potentially induce hazards.

Direct attacks related to the general sensor node vulnerability, *i.e.*, limited resources, are varied. They can supplement the SPD specification when resource availability is critical for the right operation of the sensor or its countermeasures.

If three assets (DTO.NodePowerRes, DTO.NodeProcesRes, DTO.NodeTransmRes) are attacked by one intruder (SNA.HighPotIntruder) in the same way, this elementary problem can be expressed by one threat countered by one security objective:

TDA.LimitResour. Exploiting vulnerability related to the node limited resources [Dparam <= DTO.NodePowerRes, DTO.NodeProcesRes, DTO.NodeTransmRes], an intruder [Sparam <= SNA.HighPotIntruder] causes misbehaviour, disconnection or faults within the system.
  OAVB.ResUnderControl. Control resource [Dparam <= DTO.NodePowerRes, DTO.NodeProcesRes, DTO.NodeTransmRes].

If each asset is threatened by an intruder using different methods, iterations are more convenient (please note that this issue presents an example of iteration). The considered threat and the objective which counters the threat are iterated three times for any kind of “limited resources”:

TDA.LimitResour(1). Exploiting vulnerability related to the node limited resources [Dparam <= DTO.NodePowerRes], an intruder [Sparam <= SNA.HighPotIntruder] causes misbehaviour, disconnection or faults within the system.
  OAVB.ResUnderControl(1). Control resource [Dparam <= DTO.NodePowerRes].
TDA.LimitResour(2). Exploiting vulnerability related to the node limited resources [Dparam <= DTO.NodeProcesRes], an intruder [Sparam <= SNA.HighPotIntruder] causes misbehaviour, disconnection or faults within the system.
  OAVB.ResUnderControl(2). Control resource [Dparam <= DTO.NodeProcesRes].
TDA.LimitResour(3). Exploiting vulnerability related to the node limited resources [Dparam <= DTO.NodeTransmRes], an intruder [Sparam <= SNA.HighPotIntruder] causes misbehaviour, disconnection or faults within the system.
  OAVB.ResUnderControl(3). Control resource [Dparam <= DTO.NodeTransmRes].

Direct attacks against the security-related data concerning the sensor node identity lead to counterfeit the sensor network. Some nodes disappeared, some are cloned. Applying a unique and properly controlled identifier for any node can be helpful (please note the DTO.SensorID asset). For some classes of sensors, e.g., motion sensors of tachographs, unique identifiers are used. They are controlled and inserted by dedicated PKI-based (Public Key Infrastructure) mechanisms [17]. The WSN-specific threats and measures discussed in [5] are expressed below.

TDA.CloneNode. Attacker [Sparam <= SNA.HighPotIntruder] duplicates an operational node causing that both nodes (*i.e.*, original and duplicated one of the same identity) are able to communicate with the given node.
TDA.ReplaceNode. Attacker [Sparam <= SNA.HighPotIntruder] steals (disables) operational nodes and replaces them with the malicious ones of falsified identities.
TDA.FabricateNode. Attacker [Sparam <= SNA.HighPotIntruder] adds a malicious node with fabricated identity to the network as an operational node.
TDA.SybilAttack. Attacker [Sparam <= SNA.HighPotIntruder] adds a malicious node presenting multiple identities, as if it were multiple nodes to control a considerable part of the system, breaching its redundancy.
  OIDA.ControlID. Using the properly managed unique identifiers of sensors [Dparam <= DTO.SensorID].
  OINT.IdentCapVsNodeCap. Testing limited resource (radio communication capability). Assuming that a device can access only one radio channel at a time and checking that each identity has no less capability than a physical node (all identities have channels assigned and must send messages through them simultaneously; the system detects the attack when it receives no message in its channel).
  OCON.CryptoScheme. Applying the cryptographic scheme (key management, operations) with respect to the existing communication resources.
  OEIT.IdentPositionVsNodePosition. Assuming that no identities are at the same position, checking the identity position versus the node position claiming this identity. Sensor measurements are credible when they can be associated with their physical locations.

Other security-related data include secrets or cryptographic variables [1]/Part 2. Specific attacks against them can be expressed as follows:

TDA.PowAnalys. The power consumption of some microprocessors causes leakage of information during certain cryptographic operations. The attacker [Sparam <= SNA.HighPotIntruder] uses this information to substantially reduce the key space that needs to be considered in a brute-force search for the secret key [Dparam <= DTO.CryptoKey].
  OINT.PowAnalResist. Solutions resistant to the simple/differential power analysis attacks (SPA/DPA) are implemented.
TDA.SecDataLeakage. The attacker [Sparam <= SNA.HighPotIntruder] causes that security sensitive data (authentication data, keys, credentials, *etc.*) [Dparam <= DTO.CryptoKey, DTO.RndNumber] leak out from the TOE.
  OCON.CryptoBoundary. Setup the cryptographic boundary inside the TOE from where security sensitive data [Dparam] shall not leak. The boundary encompasses the TOE parts where security sensitive data are generated, stored, updated and used.
  OCON.SecDataProt. When a node is turned off, no security material (such as a shared secret or a static public/private key) [Dparam] should have to be stored permanently in the non-volatile memory of the node (a pre-configured shared secret obviously does not satisfy this requirement).
  Refinement: See details in [10].

Attacks against node integrity encompass different tampering cases. Protection relies on tamper-resistance, fault tolerance and right management.

TDA.Tamper. Intruder [Sparam <= SNA.HighPotIntrud] physically/logically compromises a sensor node to get assets [Dparam] or disrupt the node operation [Dparam].
TDA.EnvironAttack. Intruders [Sparam <= SNA.HighPotIntrud] could compromise the sensor security through environmental attacks (thermal, electromagnetic, optical, chemical, mechanical).
TDA.PowerSupply. Users or intruders [Sparam <= SAU.IntellSensorUser, SNA.HighPotIntrud] defeat the sensor security by modifying (cutting, reducing, increasing) its power supply [Dparam <= DTO.NodePowerRes].
  OINT.TamperResistance. The TOE guarantees its own physical/logical integrity. The means of detecting physical tampering must be provided (e.g., seals, tampering detection, special reinforced cases, intrinsically safe solutions).
  OAVB.Reliability. The sensor must provide reliable service. Applying fault tolerance methods and techniques.
  OAVB.RedundNodes. Apply redundant sensor nodes, allowing to lose nodes without any impact on the network (or network application) behaviour as a whole.
  OSMN.RegularInpections. The sensor must be periodically inspected and calibrated (if necessary).

Sensor networks, similarly to other IT systems, need proper management based on proven procedures. Attacks related to insufficient administration may cause different security problems.

TIT.SecDataAdmin. Insufficient administration of the network and security related data [Dparam] of the sensor network by [Sparam <= SAU.SensorNetAdmin].
  OSMN.SecDatManag. Periodic changes to security data [Dparam] managed by [Sparam].
  OSMN.NetAdmin. Network administration and security policy procedures implementation.

Sensors may work in varied environments (industry, university laboratories, battle fields, mines, buildings, hospitals, *etc*.). They are open to unforeseen natural catastrophes, emergencies, failures, *etc*. These issues can be expressed by a simple generic which can be refined according to the circumstances specific to the operational environment.

TDA.Faults. Faults caused by [Sparam <= SNH.ForceMajeure] in hardware, software, communication procedures could place the sensor node in unforeseen conditions compromising its security.
  OINT.TamperResistance. The TOE guarantees its own physical/logical integrity. The means of detecting physical tampering must be provided (e.g., seals, tampering detection, special reinforced cases, intrinsically safe solutions).
  OAVB.RedundNodes. Apply redundant sensor nodes, allowing to lose nodes without any impact on the network (or network application) behaviour as a whole.

Depending on the sensor type and its development-, manufacturing- and maintenance processes, some threats should be specified as TPH items for the sites where these processes are run. It is important because any vulnerabilities or insufficiencies related to the processes may later influence sensors in the operational environment. To simplify this issue, all these threats are countered by implementing the right management system in the site [39]. Details depend on SARs included in the claimed EAL (not discussed here).

TPH.Design. Users or intruders [Sparam <= SAU.Developer, SAU.ManufPers, SAU.ServicePers, SNA.IndustIntrud] could try to gain illicit knowledge of the design [Dparam <= DAP.DesignData] either from the manufacturer's materials (through theft, bribery, illegal access to IT resources) or from reverse engineering.
TPH.Test. The use of non-invalidated test modes by [Sparam <= SAU.Developer, SAU.ManufPers, SAU.ServicePers] or not detected backdoors could compromise the sensor security.
TPH.SecurityData. Users or intruders [Sparam <= SAU.ManufPers, SNA.HighPotIntrud] could try to gain illicit knowledge of security data [Dparam] during security data generation, transport or storage in the equipment.
TPH.Software. Users or intruders [Sparam <= SAU.Developer, SAU.ManufPers, SAU.ServicePers, SNA.HighPotIntrud] could try to modify the sensor software.
  OSMN.SiteProcess. Site processes encompassing the development-, manufacturing- and maintenance activities in the life cycle are properly defined, implemented and managed.

#### 5.2.4. Organizational security policies (OSP) related to the intelligent sensor with respect to the full life cycle

Usually the security problem definition is expressed in a preferable way, *i.e.*, as “the protection against identified threats”, allowing to obtain more precise and coherent specifications. For this reason OSP declarations may have auxiliary meaning. For the considered sensors working in the network environment, OSPs can be used to claim general rules concerning the system usage, for example:

PAVB.SensorSysMain. The data [Dparam <= DTO.SensorData] transmitted by the sensor must be available to the supervising- or data sampling unit [Dparam] so as to allow the unit to determine fully and accurately the sampled data.
  OAVB.SensorSysMain. The data [Dparam <= DTO.SensorData], sampled by the sensor-based acquisition/monitoring system [Dparam] checked by control authorities, must be available and reflect fully and accurately the system objectives.

The second Dparam parameter of the PAVB and OAVB items can be substituted by: DIT.BaseStation, DIT.SampledDataBase or DIT.CentralUnit, with respect to the situation. Another kind of OSP applications can be conformance declarations with technical or security standards, legal acts, their specific parts or selected policies. For example, they can deal with the security management, cryptography usage, functional safety, ATEX (*fr. Atmosphere Explosible*) directive, cyber security, *etc*. Security objectives enforcing these OSPs should add more details according to given circumstances and needs. From these policies, similar security objectives for the TOE environment are usually derived. Examples of these OSP declarations are:

PSMN.ISO27001. Within the sensor development and manufacturing environment the ISMS (Information Security Management System), according to ISO/IEC 27001, should be implemented.
PSMN.ThreatVulnNotif. Notification of threats and vulnerabilities according to ISO/IEC 27001/A12.6.1. Appropriate authorities shall be immediately notified of any threats or vulnerabilities impacting systems that process their data.
Refinement: With respect to WSN and the implemented network application.
PSMN.ISA99. Ensure compliance with the ISA 99 Manufacturing and Control Systems Security Standard.
PINT.IntrinsicSafe. The intelligent sensor should be intrinsically safe.

#### 5.2.5. Assumptions related to the intelligent sensor with respect to the full life cycle

The efficacy of the countering threats and the efficacy of the enforcing OSPs depend on satisfying some assumptions addressed to the TOE environment. Below some examples of assumptions dealing with the personnel and organizational issues [21] are shown. Assumptions related to the right administration and usage supplement this set. All of them are transformed to the appropriate security objectives for the environment, which upheld these assumptions. The contents of these objectives will be reflected later, during the TOE development process, in documentation and procedures related to the development, manufacturing and maintenance processes of the discussed device. To simplify this issue, only one security objective of the TOE environment was declared, which upheld all these assumptions.

APR.Development. Sensor developers [Sparam <= SAU.Developer] must ensure that the assignment of responsibilities during the development is done in a manner which maintains IT security.
APR.Manufacturing. Sensor manufacturers [Sparam <= SAU.ManufPers] must ensure that the assignment of responsibilities during manufacturing is done in a manner which maintains IT security, and that during the manufacturing process the sensor is protected against physical attacks which might compromise IT security. All testing facilities for the manufacturing phase (test points, commands) should be removed or disabled before delivery.
APR.Delivery. Sensor manufacturers, fitters, workshops [Sparam <= SAU.ManufPers, SAU.ServicePers] must ensure that handling of the sensor is done in a manner which maintains IT security.
APR.SecDataGenAlgor. Security data generation algorithms must be accessible to authorized and trusted persons only [Sparam <= SAU.Developer, SAU.ManufPers].
APR.SecDataInsert. Security data [Dparam] must be generated, transported and inserted into the sensor in such a way to preserve its appropriate confidentiality and integrity by the authorized [Sparam <= SAU.ManufPers].
APR.ApprovedWorkshops. Installation, calibration and repair of the sensor and its monitoring unit must be carried out by trusted and approved fitters or workshops by the authorized [Sparam <= SAU.ServicePers].
APR.SoftwareUpgAnal. Software revisions must be granted security certification before they can be implemented in the sensor. There is no way to analyze or debug software in the field.
  OSMN.SiteProcess. Site processes encompassing the development-, manufacturing- and maintenance-activities in the life cycle are properly defined, implemented and managed.
APR.TrustAdmin. One or more authorized administrators [Sparm] are assigned who are competent to manage the TOE and the security of the information it contains, and who can be trusted not to deliberately abuse their privileges so as to undermine security.
  OSMN.SecManAdmin. The TOE will provide facilities to enable an authorized administrator [Sparam] to effectively manage the TOE and its security functions, and will ensure that only authorized administrators are able to access such functionality.
APR.IntendedUse. The TOE will be used to perform a task or function for which it was designed by [Sparam].
  OSMN.UserAwarn. User awareness, proper operation regulations and procedures.

The defined specification means have open character and can be applied as Common Criteria related security design patterns to a broad group of intelligent sensors as it was discussed in the above examples.

## 6. Evaluation Issues

First evaluation of this methodology was discussed in [21,22]. It was restricted to one kind of sensors, *i.e.*, to the motion sensors of digital tachographs, but all IT security development stages were shown and the design compliance with the [17] requirements was achieved. The presented set of specification items (CC-related patterns) was extended to other kinds of sensors and, for this reason, validations on these products are necessary. Two kinds of validation are possible:

- extensive validation on near-real projects like the above mentioned motion sensor; validation on intelligent sensors detecting methane is in progress and will be summarized in a separate paper;
- partial validation focused on the selected issues; it will be shown on the example below.

The aim of partial validation is to show if predefined CC-related design patterns can be applied in several typical design circumstances.

### 6.1. Validation example

The example deals with an intelligent medical sensor, working within the Secure Wireless Mote-Based Medical Sensor Network. It was elaborated on the basis of the paper [10] and with the use of [26,27] technical information sources. The example shows how to express key elements of the security target (ST) of the intelligent medical sensor considered as the target of evaluation (TOE), with the use of the Common Criteria related security design pattern elaborated for the generalized sensor model. For the purposes of this example let us call the considered TOE the “Intelligent, Mote-Based Medical Sensor (IMBMS)”. The example does not provide directly the selected parts of the ST, it rather discusses how to elaborate them. The discussion embraces the informal IT products descriptions included in the “ST introduction” and the issues dealing with the security problem definition and solution. The ST documents are rather extensive [24] and the full ST cannot be discussed here in details.

#### 6.1.1. Informal IT product description included in the “ST introduction”

**Figure 2.**
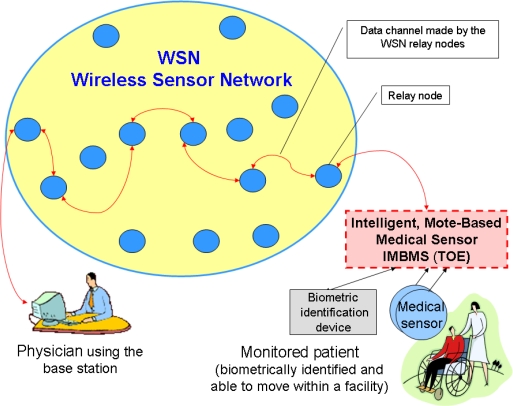
General architecture of the “Secure Wireless Mote-Based Medical Sensor Network” [10].

Apart from the TOE identifier, e.g., “IMBMS – Intelligent, Mote-Based Medical Sensor, ver. 1.0”, and the ST identifiers, e.g., “ST for the IMBMS ver. 2.0”, the “ST introduction” contains the “TOE overview” and the more detailed “TOE description”, where the basic TOE features and components are described. For the discussed IMBMS sensor, the TOE description can look as follows:

“The IMBMS system (Figure 2) is designed for distance, long-term monitoring of patients with chronic diseases. Each patient, biometrically identified, is provided with the mote with medical sensors watching her/his vital signs. Patients can move in the range of medical or home facilities. Motes are communicating with the distinguished base station through relay nodes of the wireless sensor network (WSN). Using the base station, the physician (a legitimate, authenticated user) is able to send a data query related to the given patient to the mote, which activates the appropriate medical sensors. The data channel is formed by a set of relay nodes. Many motes and base stations may exist within the system.”

**Figure 3.**
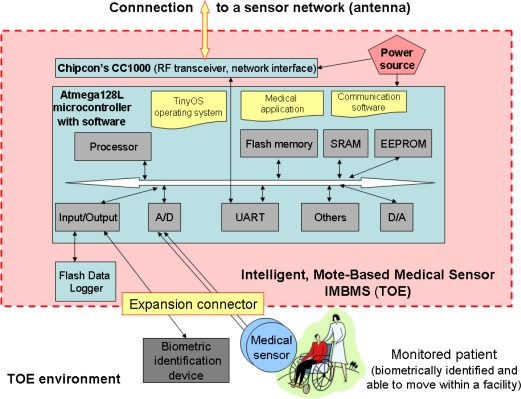
IMBMS—Intelligent Mote-Based Medical Sensor as the TOE.

One of important issues is to present what the TOE is and what is in the TOE environment, defining the TOE logical/physical components and the TOE boundary, e.g., a continuation of the “TOE description”:

“The TOE (target of evaluation) is a Crossbow MICA2 based mote (Figure 3). The TOE consists of the Atmega128L microcontroller with the TinyOS operating system, Chipcon’s CC1000 RF transceiver with antenna, “Flash Data Logger” (4-Mbit serial flash memory for storing data, measurements, user-defined information, and used for over-the-air reprogramming provided by TinyOS), power source (2 AA-type batteries), 3 LEDs displaying status, and the expansion connector. The TOE also includes the application and communication software running under TinyOS. The TOE boundary is marked with dashed lines. The medical sensors, measuring physical values related to vital signs, and the biometric identification device, like a fingerprint reader, all connected to the expansion connector, are not the parts of the TOE”.

#### 6.1.2. Protected assets

The first step of the security problem definition is the identification of assets that should be protected. Assets may be placed within the TOE or in its environment. The main asset are the patient’s medical data sampled by the mote, which can be expressed by a previously defined enhanced generic, here refined. Moreover, a new asset-type generic, *i.e.*, the TOE provided services, is defined, related to the sensor operation, *i.e.*, measuring, sampling, processing, transferring data, and displaying the mote status.

DTO.SensorData. Data measured, stored, processed, or transmitted by the intelligent sensor and data related to the network services.
Refinement: Medical data of the monitored patient.
DTO.SensorService. Services provided by a sensor: measuring, sampling, processing, transferring data, and displaying its status.

Please note that the patient’s medical data are very sensitive, e.g., they may be misused by insurance companies, their counterfeiting may cause a wrong medical diagnosis which can put the patient’s life in danger, *etc*.

The TOE also has security-related data used for the biometric identification of the monitored person. The “closest” generic which can be used is DTO.Password but it does not concern biometrics. For this reason, the previously defined set of enhanced generics is supplemented by a new item:

DTO.BioData. Biometric or physiological data used for the identification of a person.

Three standard enhanced generics related to the resource limitation are added as auxiliary but important items (patient’s monitoring should be long-term and permanent):

DTO.NodePowerRes. Energy supplying a sensor node.
DTO.NodeProcesRes. Node processing ability.
DTO.NodeTransmRes. Node data transmission ability.

Because such an IT product as IMBMS protects sensitive medical data, the evaluation assurance level (not discussed there) declared for it will be probably EAL3 or above and then the security of the development process ought to be considered as well (please note the ALC_DVS assurance family—[1]/Part 3). For this reason, all design related data should be precisely identified and expressed, e.g., with the use of the previously define item:

DAP.DesignData. Sensitive project data and documentation.
Refinement: Logical, physical design data of the TOE hardware, software specifications, code and other related documentation, development aids, test data, user data related documentation, material for software development and manufacturing process.

Careful and rigorous design, deployment and management of medical monitoring systems are very important for the patients and medical personnel.

The mote co-operates directly with the base station, which can be also distinguished as an asset.

DIT.BaseStation. A distinguished node of a wireless sensor network (WSN) used to control the network or as a gateway intermediating between WSN and other network.

#### 6.1.3. Identification of active entities—legal users and intruders

There are two specific users of IMBMS. First, the patient who uses it passively (she/he is monitored) but may unintentionally cause harmful events (disruption of the monitoring, destroying the equipment), so the patient will be considered further as an intruder. The second kind of user is the physician remotely monitoring the patient and having full access rights to the monitored data, as well as other medical personnel (a nurse) and household members taking care of the patient but having no full access rights to the monitored data. The physician is authenticated before starting the work with the base station. Both these subjects can be expressed by the iteration of the early predefined enhanced generic:

SAU.IntellSensorUser(1). Authorized entity (user, process) who/which directly or indirectly uses the intelligent sensor.
Refinement: The monitored patient.
SAU.IntellSensorUser(2). Authorized entity (user, process) who/which directly or indirectly uses the intelligent sensor.
Refinement: The medical personnel (a physician remotely monitoring patients, a nurse visiting patients) and household members.

The entire medical system, including the considered TOE, should be properly managed, developed, manufactured and maintained. For this reason, the following active entities (roles) are distinguished:

SAU.SensorNetAdmin. Authorized administrator of the sensor network and applications.
SAU.Developer. Personnel involved in the design phase (hardware/software designer, programmer, test engineer).
SAU.ManufPers. Personnel involved in the manufacturing processes (components manufacturing, assembly, security data insertion, storage, distribution, repair).
SAU.ServicePers. Personnel involved in sensor or sensors system maintenance (storage, installation, inspection, calibration, repair).

The intruder operating within the Secure Wireless Mote-Based Medical Sensor Network and the intruder operating within the development-, manufacturing- and maintenance-environment can be expressed using the previously defined generics:

SNA.HighPotIntruder. Attacker having high level skills, enough resources and deep motivation to perform a deliberate attack.
SNA.IndustIntrud. Industry spy or intruder trying to get or counterfeit the design data.

#### 6.1.4. Identification of threats and security objectives which counter them

For given threats, it is necessary to declare security objectives which counter them, for the TOE (IMBMS) and/or for its environment. Please note that this declaration expresses the responsibility in countering threats and decides about the future TOE shape and features, because the TOE built-in countermeasures (*i.e.*, security functions) are derived from the TOE security objectives only.

The key issue for the intelligent medical sensor is the possibility to compromise the mote by the intruder of high attack potential acting from the outside (WSN, base station) and exploiting vulnerabilities of the implemented network protocols. This compromising may lead to the sensor data and/or security-related data eavesdropping and/or their counterfeiting. These issues can be expressed by two predefined threat-type generics countered by four security objectives declared for the TOE only:

TDA.NodeCompromise. Attacker [Sparam <= SNA.HighPotIntrud] can: eavesdrop the traffic, inject packets or replay older messages because wireless communication generally is not secure. After the node compromising, all information it holds [Dparam <= DTO.SensorData, DTO.BioData, DIT.BaseStation] is known to the attacker and/or node operation or communication is broken [Dparam <= DTO.SensorService].
TDA.SensDataMdfy. Intruder [Sparam <= SNA.HighPotIntruder] modifies sensor data (falsifies them) [Dparam <= DTO.SensorData, DTO.BioData].

Security objectives declared for the TOE:

OACC.Access. The sensor must control access of connected entities.
OIDA.Authentication. The sensor must authenticate connected entities.
OADT.Audit. The sensor must audit attempts to undermine its security and should trace them to the associated entities.
OINT.Processing The sensor must ensure that the processing of input to derive output data [Dparam <= DTO.SensorData] is accurate.

Applying a tampering attack, the intruder of high attack potential may intentionally compromise the physical or logical TOE integrity to get, modify or destroy the sensor data (any kind of data) and/or disrupt the intelligent sensor operation. Additionally, the monitored patient may unintentionally damage the mote or disrupt the sensor operation, which may be considered as tampering too. This threat is expressed by one enhanced generic countered by two objectives for the TOE and two for its environment:

TDA.Tamper. Intruder [Sparam <= SNA.HighPotIntrud, SAU.IntellSensorUser(1)] physically/logically compromises a sensor node to get assets [Dparam <= DTO.SensorData, DTO.BioData] or disrupt the node operation [Dparam <= DTO.SensorService].

Security objectives declared for the TOE:

OINT.TamperResistance. The TOE guarantees its own physical/logical integrity. The means of detecting physical tampering must be provided (e.g., seals, tampering detection, special reinforced cases, intrinsically safe solutions).
OAVB.Reliability. The sensor must provide reliable service. Applying fault tolerance methods and techniques.

Security objectives declared for the TOE environment (the second was specially defined):

OSMN.RegularInpections. The sensor must be periodically inspected and calibrated (if necessary).
OEPH.PatientSecurity. The monitored patient is within the access-restricted area and the medical personnel or household members take care of her/him.

The shortage of resources, especially energy, may be critical for long-term, permanent monitoring of the patient. It can be solved by remote reading of the resources status, watching equipment locally and reaction of responsible persons:

TDA.LimitResour. Exploiting vulnerability related to the node limited resources [Dparam <= DTO.NodePowerRes, DTO.NodeProcesRes, DTO.NodeTransmRes], an intruder [Sparam <= SNA.HighPotIntruder] causes misbehaviour, disconnection or faults within the system.

Security objectives declared for the TOE:

OAVB.ResUnderControl. Control resource [Dparam <= DTO.NodePowerRes, DTO.NodeProcesRes, DTO.NodeTransmRes].

Security objective declared for the TOE environment (defined):

OEPH.PatientSecurity. The monitored patient is within the access-restricted area and the medical personnel or household members take care of her/him.

Within the TOE environment there operate other motes, base stations and numerous WSN relay nodes. Each of them, if uncontrolled, may be accessible to potential intruders. This issue (problem and its solution) can be expressed as:

TIT.UncontrolledArea. Due to uncontrolled pervasiveness of a wireless sensors network, the node works in an uncontrolled network area accessible to potential intruders [Sparam <= SNA.HighPotIntruder].

Security objective declared for the TOE environment:

OEIT.SecPerimVsTrRangeCtrl. The physically controlled security perimeter, where nodes are placed, should be defined with respect to the range of wireless transmission.

The entire WSN should be properly used and managed. This issue can be expressed in the following way:

TIT.SecDataAdmin. Insufficient administration of the network and security related data [Dparam] of the sensor network by [Sparam <= SAU.SensorNetAdmin].

Security objectives declared for the TOE environment:

OSMN.SecDatManag. Periodic changes to security data [Dparam] managed by [Sparam <= SAU.SensorNetAdmin].
OSMN.NetAdmin. Network administration and security policy procedures implementation.

The design related data can be eavesdropped and abused in different ways (TPH.Design). Generally, they can be used to prepare attacks in the TOE operational environment or can be used by dishonest competitors. The second issue deals with insufficient testing (TPH.Test). All these problems can be solved by the implementation of technical, physical and organizational measures within the site where the TOE is developed, manufactured and maintained.

TPH.Design. Users or intruders [Sparam <= SAU.Developer, SAU.ManufPers, SAU.ServicePers, SNA.IndustIntrud] could try to gain illicit knowledge of the design [Dparam <= DAP.DesignData] either from the manufacturer's materials (through theft, bribery, illegal access to IT resources) or from reverse engineering.
TPH.Test. The use of non-invalidated test modes by [Sparam <= SAU.Developer, SAU.ManufPers, SAU.ServicePers] or not detected backdoors could compromise the sensor security.

Security objective declared for the TOE environment:

OSMN.SiteProcess. Site processes encompassing the development-, manufacturing- and maintenance activities in the life cycle are properly defined, implemented and managed.

#### 6.1.5. Organizational security policies (OSPs) and security objectives which enforce them

Similarly to the threats, for given OSPs the security objectives, which enforce them, should be declared for the TOE (IMBMS) and/or for its environment. In this example two OSPs are defined, covering the right operation of the system and legal compliance with HIPAA [40]. Only security objectives for the TOE environment were declared for OSPs.

PAVB.SensorSysMain. The data [Dparam <= DTO.SensorData] transmitted by the sensor must be available to the supervising- or data sampling unit [Dparam <= DIT.BaseStation] so as to allow the unit to determine fully and accurately the sampled data.

Security objective enforcing the OSP within the TOE environment:

OAVB.SensorSysMain. The data [Dparam <= DTO.SensorData], sampled by the sensor-based acquisition/monitoring system [Dparam <= DIT.BaseStation] checked by control authorities, must be available and reflect fully and accurately the system objectives.
PSMN.HIPAA. Ensure the compliance with the Health Insurance Portability and Accountability Act.

Security objective enforcing the OSP within the TOE environment:

OEPH.HIPAA. The medical system should comply with the HIPAA Act.

#### 6.1.6. Assumptions and TOE environment security objectives which uphold them

The first group of assumptions concerns the development-, manufacturing- and maintenance environment (called also the site) and the second deals with the right management and intentional use of the TOE in the operational environment. The TOE environment security objectives which uphold these assumptions were declared:

APR.Development. Sensor developers [Sparam <= SAU.Developer] must ensure that the assignment of responsibilities during the development is done in a manner which maintains IT security.
APR.Manufacturing. Sensor manufacturers [Sparam <= SAU.ManufPers] must ensure that the assignment of responsibilities during manufacturing is done in a manner which maintains IT security, and that during the manufacturing process the sensor is protected against physical attacks which might compromise IT security. All testing facilities for the manufacturing phase (test points, commands) should be removed or disabled before delivery.
APR.Delivery. Sensor manufacturers, fitters, workshops [Sparam <= SAU.ManufPers, SAU.ServicePers] must ensure that handling of the sensor is done in a manner which maintains IT security.
APR.ApprovedWorkshops. Installation, calibration and repair of the sensor and its monitoring unit must be carried out by trusted and approved fitters or workshops by the authorized [Sparam <= SAU.ServicePers].

This security objective upholds assumptions within the TOE environment:

OSMN.SiteProcess. Site processes encompassing the development-, manufacturing- and maintenance activities in the life cycle are properly defined, implemented and managed.
APR.TrustAdmin. One or more authorized administrators [Sparm <= SAU.SensorNetAdmin] are assigned who are competent to manage the TOE and the security of the information it contains, and who can be trusted not to deliberately abuse their privileges so as to undermine security.

This security objectives upholds assumption within the TOE environment:

OSMN.SecManAdmin. The TOE will provide facilities to enable an authorized administrator [Sparam <= SAU.SensorNetAdmin] to effectively manage the TOE and its security functions, and will ensure that only authorized administrators are able to access such functionality.
APR.IntendedUse. The TOE will be used to perform a task or function for which it was designed by [Sparam <= SAU.IntellSensorUser(2)].

This security objective upholds assumption within the TOE environment:

OSMN.UserAwarn. User awareness, proper operation regulations and procedures.

### 6.2. Summary of the example

The first summary issue concerns the next steps of the IT security development process, not discussed in the paper. The security objectives for the TOE will be expressed by the security functional requirements [1]/Part 2, implemented in the security functions within the TOE at the claimed EAL, and finally evaluated. Seven TOE security objectives (all countering threats) are identified. Around these issues the future security functions will be developed. They are:

OACC.Access, OIDA.Authentication, OADT.Audit, OINT.Processing, OINT.TamperResistance, OAVB.Reliability, OAVB.ResUnderControl.

The security objectives for the TOE environment, despite of their destination (upholding assumptions, supporting TOE in countering threats or enforcing OSPs), will be envisaged in the different kinds of the development-, manufacturing- and operational-documentation and in security mechanisms implemented in the TOE environment. During the evaluation they are considered as dogmas. For IMBMS ten objectives for the TOE environment were identified:

OSMN.RegularInpections, OEPH.PatientSecurity, OEIT.SecPerimVsTrRangeCtrl, OSMN.SecDatManag, OSMN.NetAdmin, OSMN.SiteProcess, OAVB.SensorSysMain, OEPH.HIPAA, OSMN.SecManAdmin, OSMN.UserAwarn.

The second summary issue concerns Common Criteria related security design patterns. The simplified validation of the specification means (design patterns) elaborated for intelligent sensors shows that all basic security issues of the representative intelligent medical sensor can be expressed this way. As a result of this validation, this set was enriched by five new enhanced generics expressing specific issues. The first concerns biometrics, the second sensors services, while others are specific for medical systems:

DTO.BioData, DTO.SensorService, OEPH.PatientSecurity, PSMN.HIPAA, OEPH. HIPAA.

## 7. Conclusions

The paper shows how to apply the Common Criteria methodology to the process of intelligent sensor development. Here presented Common Criteria related security design patterns can be applied in a broader group of sensors and their applications. One representative intelligent sensors example and its applications were analyzed and then a generalized TOE model was developed. For this model the set of specification means as CC-related design patterns was defined to express security features of a broad range of sensors and their network applications. The capabilities of this set of design patterns were validated on the intelligent mote-based medical sensor.

The intelligent sensor development can be discussed as one of emerging domains of the Common Criteria standard application. The contribution of the paper is to support IT security developers by providing them with:

- Common Criteria related security design patterns, called here enhanced generics, to define elementary security problems (*i.e.*, threats, security organizational policies, assumptions) and elementary solutions of these problems (*i.e.*, security objectives), with respect to the intelligent sensors needs and the life-cycle model,
- knowledge how to apply these patterns to elaborate the Common Criteria complaint security model, called security target.

The achieved results will be used to extend the author’s elaborated knowledge base supporting IT security developers. For a given intelligent sensor project the specification means can be selected from the proposed set and refined to describe more precisely and adequately the TOE security issues. The issues presented here encompass two most difficult IT security development stages with respect to specific needs of the sensors. General knowledge about the Common Criteria methodology is needed to express the next stages, *i.e.*, elaboration of the security functional requirements on the security objectives basis, elaboration of the security functions using these requirements, as well as the elaboration of evaluation evidences.

Intelligent sensors are a broad and varied class of devices. For this reason, validations on different kinds of designs should be performed to improve the defined patterns. The validation on the motion sensor design has been finished recently [21,22], while the validation on the intelligent sensors detecting methane in the mining environment is in progress. Two variants are considered:

- sensor operating within a wireless network,
- sensor supervised by a monitoring unit and connected through a copper cable.

The mentioned validations will be supported with the elaborated knowledge engineering tool [36,37] based on Protégé [38]. While the CC-related design patterns were defined, some problems concerning common understanding of terms, their precise definitions, right semantics, and relations with other terms are encountered. This can be solved by the ontology definition. The elaboration of this ontology can be considered as another direction of the planned works. This ontology should express a general attack model, encompassing both simple and complex attacks. The latter should be able to consider the primary cause of the attack being the leverage for other attack or series of attacks. This way the propagation and escalation effects, which may occur in wireless sensors networks, can be taken into account.

Please note that the Common Criteria methodology supplements, organizes and puts in order the activities and best practices of IT products or systems developers. Thanks to the CC methodology, sensor systems developers, manufacturers and users can provide assurance to their solutions. Besides, the methodology allows independent evaluation and worldwide recognized certification.
